# First bioelectronic immunoplatform for quantitative secretomic analysis of total and metastasis-driven glycosylated haptoglobin

**DOI:** 10.1007/s00216-022-04397-6

**Published:** 2022-11-08

**Authors:** Cristina Muñoz-San Martín, Ana Montero-Calle, María Garranzo-Asensio, Maria Gamella, Víctor Pérez-Ginés, María Pedrero, José M. Pingarrón, Rodrigo Barderas, Noemí de-los-Santos-Álvarez, María Jesús Lobo-Castañón, Susana Campuzano

**Affiliations:** 1 Departamento de Química Analítica, Facultad de CC. Químicas, Universidad Complutense de Madrid, 28040 Madrid, Spain; 2 UFIEC, Instituto de Salud Carlos III, 28220 Majadahonda, Madrid, Spain; 3 Departamento de Química Física y Analítica, Universidad de Oviedo, 33006 Oviedo, Spain; 4 Instituto de Investigación Sanitaria del Principado de Asturias, 33011 Oviedo, Spain

**Keywords:** Glycosylated haptoglobin, Amperometry, Multiplexed immunoplatform, Secretome, Metastatic CRC cells

## Abstract

The glycosylation status of proteins is increasingly used as biomarker to improve the reliability in the diagnosis and prognosis of diseases as relevant as cancer. This feeds the need for tools that allow its simple and reliable analysis and are compatible with applicability in the clinic. With this objective in mind, this work reports the first bioelectronic immunoplatforms described to date for the determination of glycosylated haptoglobin (Hp) and the simultaneous determination of total and glycosylated Hp. The bioelectronic immunoplatform is based on the implementation of non-competitive bioassays using two different antibodies or an antibody and a lectin on the surface of commercial magnetic microcarriers. The resulting bioconjugates are labeled with the horseradish peroxidase (HRP) enzyme, and after their magnetic capture on disposable electroplatforms, the amperometric transduction using the H_2_O_2_/hydroquinone (HQ) system allows the single or multiple detection. The developed immunoplatform achieves limits of detection (LODs) of 0.07 and 0.46 ng mL^−1^ for total and glycosylated Hp in buffer solution, respectively. The immunoplatform allows accurate determination using simple and relatively short protocols (approx. 75 min) of total and glycosylated Hp in the secretomes of in vitro–cultured colorectal cancer (CRC) cells with different metastatic potentials, which is not feasible, due to lack of sensitivity, by means of some commercial ELISA kits and Western blot methodology.

**Graphical abstract:**

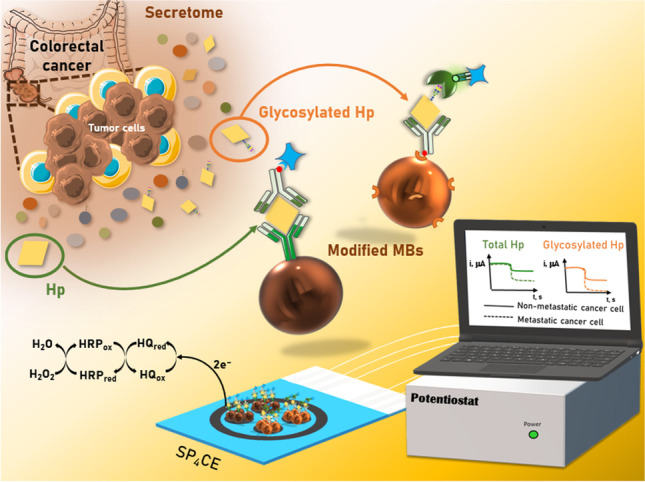

**Supplementary Information:**

The online version contains supplementary material available at 10.1007/s00216-022-04397-6.

## Introduction

In addition to abundant alterations in the proteome, post-translational modifications (PTMs) of proteins deserve attention in early detection of diseases and their monitoring. Glycosylation is one of the most important PTMs and plays a critical role in various biological processes, such as protein function and cell-cell interaction, which makes it as an important biomarker for cancer and infectious diseases [1–3].

Glycosylation is known to be associated with tumor progression and metastasis of several types of cancer. Glycan structure is often terminated by sialic acids (SA): a group of monosaccharides synthesized in animals comprising 50 species of nine-carbon neuraminic acids. Due to their negative electrical charge, SA act as “bridging” molecules between cells and between cells and the extracellular matrix. In this way, they function as important modulators for cell-cell interaction and influence the fate and behaviors of these cells [4]. To improve the specificity and sensitivity of diagnosis, some studies have been directed toward the in-depth characterization of glycosylation of specific target glycoproteins [3, 5]. It is important to note that direct analysis of intact glycoproteins, rather than glycan profiling, which is still considered the gold standard for its high throughput and sensitivity, accounts advantageous because of its simplicity, ease of sample preparation, and analysis time saving. In addition, glycoprotein interrogation has important advantages over other biomolecules as disease markers, since glycan biosynthesis is more readily affected by disease states than protein production, and aberrant glycosylation affects almost all glycoproteins produced in the diseased cell [2, 6].

Among glycoproteins, haptoglobin (Hp), a 105-kDa tetrameric molecule that accounts for 0.4 to 2.6% of total blood proteins [6], should be highlighted. It is a positive acute-phase response protein that acts as an immunomodulator [7] and is mainly secreted by the liver but also by the lungs and skin [2, 5]. Hp, circulating in biofluids such as human serum and plasma at levels of milligrams per milliliter, performs important and multiple functions. On the one hand, it has an antioxidant role by decreasing the oxidative and toxic effect of free hemoglobin (Hb) in plasma. Free Hb binds rapidly and with high affinity to Hp [8], and the Hb-Hp complex is rapidly cleared from plasma by monocytes and macrophages. On the other hand, Hp stimulates angiogenesis and plays an important role in several processes of the immune response, reducing the severity of autoimmune inflammatory processes [2, 5]. Hp is an acidic highly sialylated glycoprotein with four N-glycosylation sites in its β-chain (Asn184, 207, 211, and 241) [6, 7], which has gained considerable attention due to its potential as a signature molecule that exhibits aberrant glycosylation in inflammatory disorders and various forms of malignant neoplasms (gastric, pancreatic, hepatic, prostate, lung, breast, ovarian, and colon) [2–4, 6, 8, 9]. Owing to this, the study of the glycosylation status of Hp is a major target in cancer research particularly interesting for prognostic and diagnostic purposes [2, 5].

Currently, glycosylation alterations of glycoproteins are monitored mainly using omics-, lectin- [10, 11], and mass spectrometry (MS)–based technologies [6, 7, 9, 12]. Among them, MS is widely used, and it is considered one of the most precise techniques for qualitative and quantitative analyses of glycosylation [6]. However, it shows limitations for its implementation in the clinic due to the cost and multiple steps required for sample preparation, the difficulties of analysis and the complex interpretation of the data [2, 3]. Therefore, it would be ideal to have tools for the rapid, easy, and accurate determination of glycosylated biomarkers more compatible with their use in the clinic.

In this context, advances in the design and performance of electrochemical biosensors consolidate them as promising bioanalytical tools, at the forefront of modern techniques for the determination of clinical biomarkers. Several attributes render electrochemical biosensors well suited for clinical analysis, keeping alive the hope of extending their use from specialized laboratories to public settings, including hospital point-of-care testing (POCT), outpatient, or even home environments. First, they are built on substrates easily miniaturized and manufactured in batches. Second, they need simple, portable, and low-cost peripheral instruments with low power consumption. Third, they are highly selective and sensitive, with fast response time, ease of use, and low cost. Fourth, they allow the analysis of complex, turbid, and/or colored samples at both multiplexed and multiomics levels. Finally, they are compatible with complex centralized electrochemical instrumentation or with simple detection devices that can be operated by any user in any environment, even at home, such as glucometer-type devices. It is important to note that, nowadays, the reported biosensors for the determination of glycosylated biomarkers mainly use aptamers [13, 14] or lectins [15, 16] as recognition elements. Moreover, although some electrochemical biodevices have been reported for the determination of total Hp [17–21], to date, no electrochemical immunoplatform has been described for the determination of glycosylated Hp or for total and glycosylated Hp simultaneous determination.

On the other hand, the analysis of secretomes of cancer cells or tissues is currently considered of great interest for the identification of biomarkers, the clarification of carcinogenesis mechanisms, and improved diagnosis and treatment monitoring. Secretome analysis offers an ideal alternative to narrow down the list of candidate biomarkers quickly and efficiently, most likely to be successful in validation testing with valuable clinical samples [22]. The secretome is among the multitude of factors affecting the tumor microenvironment of accepted relevance to the development and metastasis of cancer cells [23]. Cancer cell secretomes harbor secreted proteins, including growth factors, proteases, cell motility factors, cytokines, chemokines, and/or cell surface receptors. Some of these proteins play pivotal roles in tumor progression, invasion, metastasis, and/or angiogenesis by regulating cell-to-cell and cell-to-extracellular matrix interactions. Furthermore, they are likely to be found in body fluids, such as blood or urine, and therefore can be measured by non-invasive diagnostic tests and help identify potential serum tumor biomarkers [22, 24].

Considering all this background, we report in this work the first bioelectronic immunoplatform that allows the single or multiplexed determination of total and glycosylated Hp. The bioplatform uses disposable electrodes, is assisted by magnetic micro supports, and involves non-competitive immunoassays using capture and detector bioreceptors of the same (antibody-antibody) or different (antibody-lectin) nature for the determination of total and glycosylated Hp, respectively. The immunoplatform exhibits suitable analytical and operational characteristics that allow the quantitative analysis of the secretome of CRC cells with different metastatic potentials.

## Experimental section

### Apparatus and electrodes

Single amperometric measurements were performed with a CHI1140A potentiostat while multiple amperometric measurements were carried out with a CHI1030B multichannel potentiostat (CH Instruments, Inc.). Single (DRP-110, SPCE), dual (DRP-C1110, SPdCE), and quadruple (DRP-4W110, SP_4_CE) screen-printed carbon electrodes (SPCEs) and the corresponding connector cables (DRP-CAC, DRP-BICAC, and DRPCONNECT4W, respectively) were provided by Metrohm-Dropsens S.L. A pH meter (Basic 20+, Crison), a Vortex (Velp Scientifica), a BioSan TS-100 thermo shaker for microtubes (Thermo), a magnetic stirrer (Inbea S.L.), and a DynaMag™-2 magnet (Invitrogen-ThermoFisher Scientific) were also used. Efficient trapping of the magnetic beads (MBs) onto the SPCEs, SPdCEs, or SP_4_CEs working electrodes was achieved by using homemade polymethylmethacrylate (PMMA) casings embedding one, two, or four neodymium magnets (AIMAN GZ).

### Reagents and solutions

Carboxylic acid–modified magnetic microbeads (HOOC-MBs, *Ø* = 2.8 μm, Dynabeads® M270) and fluorescein isothiocyanate (FITC)–conjugated *Sambucus nigra* lectin isolated from elderberry bark (SNA-FITC) were provided by Invitrogen-ThermoFisher™. Neutravidin-coated magnetic particles (Neu-MBs, *Ø* = 1.0 μm) were supplied by SpeedBeads. Mouse anti-human Hp capture antibody (CAb), biotinylated goat anti-human Hp antibody (Btn-Ab), and human Hp standard were purchased as a sandwich ELISA kit (DuoSet®, DY8465-05, R&D Systems). Streptavidin-HRP complex (Strep-HRP) and Fab fragments from polyclonal anti-FITC conjugated to HRP (Fab fragments anti-FITC-HRP) were acquired from Roche Diagnostics GmbH. Blocker casein (BB, 1% w/v casein in PBS) and One Step™ Ultra 3,3′,5,5′-tetramethylbenzidine (TMB) substrate solutions were obtained from Thermo Scientific™. Sodium hydroxide (NaOH) from Labkem; NaCl, KCl, NaH_2_PO_4_×2H_2_O, and Na_2_HPO_4_ from Scharlab; Tris from Panreac; and 2-morpholinoethanesulfonic acid (MES) from Gerbu were also used. N-(3-Dimethylaminopropyl)-N′-ethylcarbodiimide (EDC), N-hydroxysulfosuccinimide (sulfo-NHS), 3-aminophenylboronic acid (APBA), hydrogen peroxide (H_2_O_2_, 30% w/v), hydroquinone (HQ), ethanolamine, human hemoglobin (Hb), immunoglobulin G from human serum (IgG), and human serum albumin (HSA) were acquired from Sigma-Aldrich. Ethylenedinitrilotetraacetic acid (EDTA) was purchased from Merck. Recombinant human interleukin-13 receptor alpha 2 (IL-13Rα2, R&D Systems), recombinant human cadherin-17 (CDH-17, OriGene Technologies, Inc.), and recombinant human tumor necrosis factor alpha (TNF-α, Pharmigen) were tested as potential interfering substances. Other solutions, prepared in deionized water from a Milli-pore Milli-Q purification system (18.2 MΩ cm), were phosphate buffer saline (PBS) consisting of 10 mM phosphate buffer solution containing 2.7 mM KCl and 137 mM NaCl, pH 7.4; 50 mM phosphate buffer, pH 6.0; 100 mM phosphate buffer, pH 8.0; 25 mM MES buffer solution, pH 5.0; and 100 mM Tris-HCl buffer, pH 7.2.

### Modification of MBs

#### Preparation of the magnetic bioconjugates for the determination of total Hp

Three microliters of the commercial HOOC-MBs suspension were incubated twice with 50 μL of MES buffer (pH 5.0) in 1.5-mL microcentrifuge tubes for 10 min under continuous shaking (25 °C, 950 rpm). After each incubation step, the MBs were placed in a magnetic concentrator for 3 min to remove the supernatant. Activation of the HOOC-MBs was then performed through their incubation with 25 μL of a mixture solution containing EDC and sulfo-NHS (50 mg mL^−1^ each prepared in MES buffer) under constant shaking (35 min, 25 °C, 950 rpm). After washing twice with 50 μL of MES buffer, the activated MBs were incubated for 15 min (25 °C, 950 rpm) with 25 μL of 25 μg mL^−1^ CAb solution prepared in MES buffer. Thereafter, the CAb-MB immunocomplexes were rinsed twice with 50 μL of MES buffer and incubated with 25 μL of a 1.0-M ethanolamine solution prepared in phosphate buffer (pH 8.0) for 1 h (25 °C, 950 rpm) to block the remaining HOOC-activated groups. After the modified MBs were washed once with 50 μL of Tris-HCl buffer (pH 7.2) and twice with 50 μL of BB, the blocked CAb-MB immunocomplexes were stored at 4 °C in sterilized PBS until use.

To perform the analysis, different working solutions containing a mixture of Hp standard (or the corresponding sample) and biotinylated detector antibody (Btn-Ab, 0.05 μg mL^−1^) prepared in BB were added to the CAb-MB immunocomplexes, incubated for 30 min (25 °C, 950 rpm) and washed twice with 50 μL of BB. Then, enzymatic labeling was performed by incubating the modified MBs with 25 μL of a 1/500-diluted Strep-HRP solution prepared in BB (30 min, 25 °C, 950 rpm). Finally, the MB immunocomplexes were washed twice with 50 μL of BB.

#### Preparation of the magnetic bioconjugates for the determination of glycosylated Hp

A 3-μL aliquot of the commercial Neu-MBs was washed twice with 50 μL of 10 mM PBS (pH 7.4) and incubated with 25 μL of 100 ng mL^−1^ Btn-Ab solution in PBS for 30 min. Unless stated otherwise, all incubation steps were carried out at 25 °C and 950 rpm and the washing steps were performed by placing the MBs for 3 min in the magnetic concentrator to remove the supernatant. Once the immunocaptors (Ab-Btn-Neu-MBs) were washed, incubation with 25 μL of the corresponding Hp or sample solution (prepared in PBS) was carried out for 30 min. The modified MBs were then washed twice with 50 μL of PBS, and the glycosylation recognition process took place by incubating the MBs with 25 μL of a 2.5-μg mL^−1^ SNA-FITC solution in PBS for 30 min. SNA lectin is a tetrameric 140-kDa glycoprotein that binds preferentially to sialic acid (SA) attached to terminal galactose in α-2,6 and to a lesser degree, α-2,3 linkage [10, 11, 25]. Once the modified MBs were washed twice with 50 μL of BB, enzymatic labeling with Fab fragments anti-FITC-HRP was performed with 25 μL of a 0.5-U mL^−1^ solution prepared in BB (15 min). To complete the preparation process, two washing steps were conducted with 50 μL of BB.

#### Amperometric measurements

MBs were resuspended in 50 μL (single determination) or 5 μL (multiplexed determinations) of 50 mM phosphate buffer (PB), pH 6.0, and magnetically trapped on the surface of the SPCE, SPdCE, or SP_4_CE working electrodes, respectively, using magnets encapsulated in the appropriate PMMA casing. The corresponding ensemble electrode/magnet holding block was connected to the potentiostat via the specific cable, and it was immersed into an electrochemical cell containing 10 (SPCE and SPdCE) or 20 (SP_4_CE) mL of 50 mM PB supplemented with 1 mM HQ. Amperometric measurements were carried out at room temperature under continuous stirring, and a detection potential of − 0.20 V was applied (vs*.* Ag pseudoreference electrode). The current variation produced after the addition of 50 μL (SPCE and SPdCE) or 100 μL (SP_4_CE) of a 100-mM H_2_O_2_ solution (prepared in 50 mM phosphate buffer, pH 6.0) was recorded. Numbers and error bars given throughout the manuscript were estimated as the mean values and three times the standard deviation (s) of three replicates (*n* = 3), respectively.

### Cell culture, and CRC cell secretomes and protein extracts

KM12C, KM12SM, and KM12L4a cells were obtained from I. Fidler’s laboratory (MD Anderson Cancer Center), whereas SW480 and SW620 cells were from the American Type Culture Collection (ATCC) [26, 27].

Cells were cultured until 90% confluence at 37 °C and 5% CO_2_ in Dulbecco’s modified Eagle’s medium (DMEM) (Lonza) supplemented with 10% fetal bovine serum (FBS) (Sigma), penicillin/streptomycin (Lonza), and l-glutamine (Lonza).

To obtain the secretomes, cells were washed three times with Dulbecco’s phosphate-buffered saline solution (DPBS, Gibco). Then, cells were incubated for 1 h in starving conditions (DMEM supplemented with penicillin/streptomycin and glutamine without FBS) at 37 °C and 5% CO_2_. Next, cells were washed with DPBS three times, and subsequently incubated for a total of 48 h in starving conditions at 37 °C and 5% CO_2_. Finally, conditioned media (secretome) was collected and clarified for 5 min at 180 g to remove any cell debris.

To obtain protein extracts, the above-prepared cells were washed with DPBS and harvested using 4 mM EDTA-PBS. After centrifugation at 180 g for 5 min, the cell pellets were lysed by mechanical disaggregation through 25-G syringes using 1 mL of RIPA buffer (Sigma) supplemented with phosphatase and protease inhibitors (MedChemExpress). The supernatant containing the protein extracts was collected after centrifugation at 12000 g for 15 min. Protein concentration was calculated using the Trp method [28].

Secretomes and protein extracts were analyzed using the developed immunoplatforms and Western blot (WB) methodology.

### Western blot

Ten micrograms of KM12C, KM12SM, KM12L4a, SW480, and SW620 protein extracts was separated on 10% SDS-PAGE under reducing conditions and transferred to nitrocellulose membranes (100 V, 90 min). The membranes were blocked for 1 h using 3% BSA in 0.1% Tween-PBS, and alternatively incubated overnight at 4 °C with the biotinylated goat anti-human Hp antibody (Btn-Ab, 1/1000, component of the DuoSet®, DY8465-05, R&D Systems) or anti-RhoGDi antibody (1/1000; Santa Cruz Biotechnology) in the same solution. After three washes with 0.1% Tween PBS (PBST), the membranes were incubated with either 1/500 Strep-HRP (RayBiotech) or 1/1000 HRP-anti mouse IgG (Sigma), respectively, for 1 h at room temperature. The membranes were then washed thrice with PBST, and a luminescence signal was developed with the ECL Pico Plus chemiluminescent reagent (Thermo Fisher Scientific) and detected on an Amersham Imager 680 (GE Healthcare).

The intensity of each WB lane was qualitatively measured using ImageJ. Hp and RhoGDi protein bands were normalized using the loading control (RhoGDi).

## Results and discussion

**Fig. 1:**
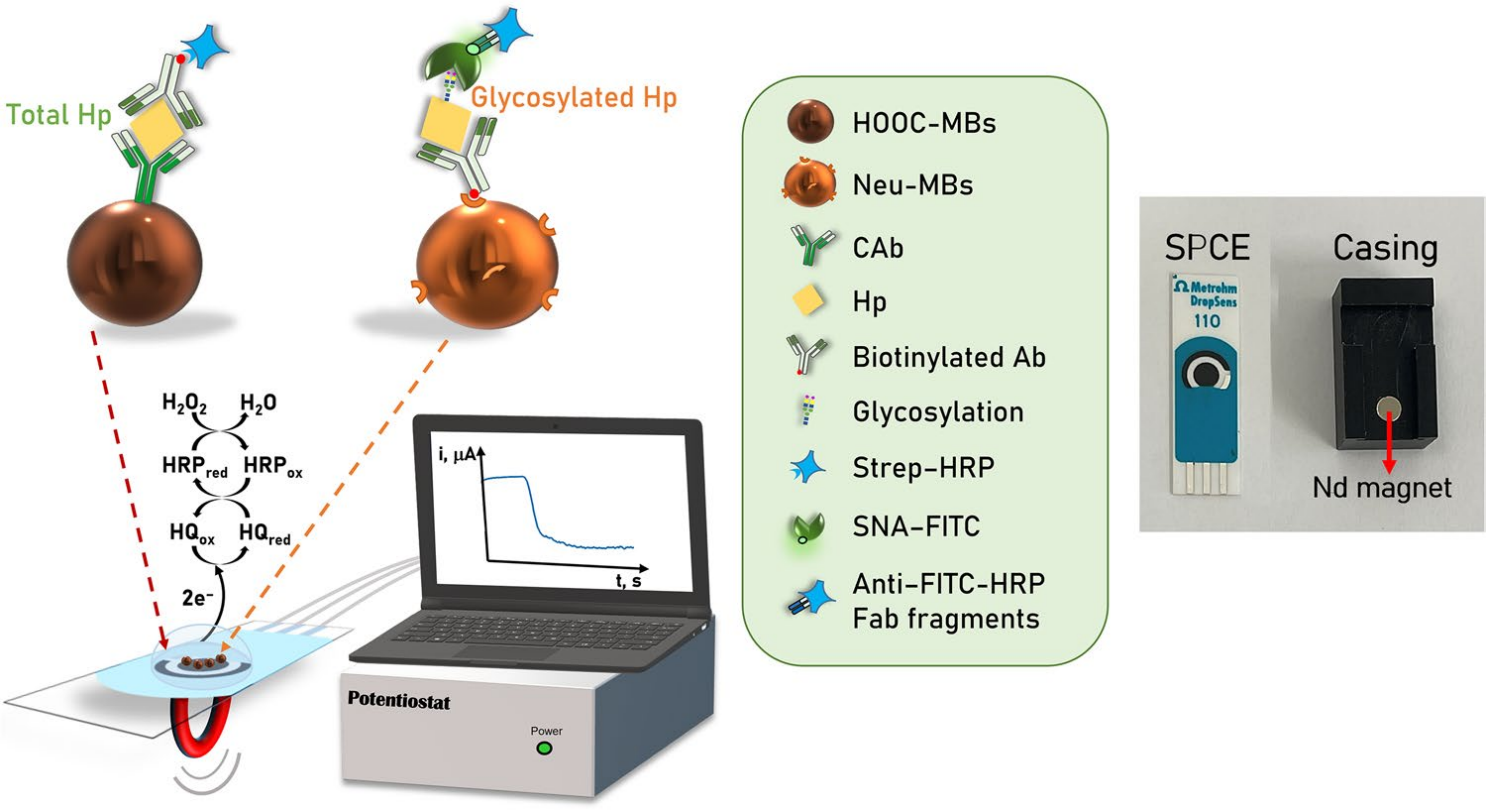
MB-assisted immunoplatform for the amperometric determination of total and glycosylated Hp

In this work, the first bioelectronic immunoplatform reported to date for the single or simultaneous determination of total and/or glycosylated Hp is proposed. The immunoplatform involves the use of solid microcarriers (magnetic microparticles, MBs) for the implementation of bioassays as well as amperometric transduction at screen-printed carbon electrodes (SPCEs) for single or multiple detection. As shown in Fig. 1, the determination of both targets is performed using non-competitive immunoassay formats. While the determination of total Hp employs a capture antibody (CAb) covalently immobilized on the surface of HOOC-MBs and enzymatically labeled (with a commercial Strep-HRP conjugate) biotinylated detector antibody (Btn-Ab), the determination of glycosylated Hp involves the immobilization of the same biotinylated antibody (Btn-Ab) on the surface of neutravidin-modified MBs (Neu-MBs), and the recognition of the sialylated regions of the glycosylated target protein using SNA-FITC, which is enzymatically labeled with anti-FITC-HRP Fab fragments.

In both cases, the resulting magnetic bioconjugates were captured on screen-printed carbon electrodes for single (SPCEs) or multiplexed (SP_4_CEs) transduction, which were previously placed in appropriate homemade casings to ensure their reproducible and stable magnetic capture. Amperometric transduction was performed in stirred solutions using the HRP/HQ/H_2_O_2_ system, resulting in a variation in the cathodic current which was directly proportional to the concentration of the target protein.

### Immunoplatforms for the single determination of total and glycosylated Hp

The experimental variables involved in the preparation of the immunoplatforms for the single determination of total and glycosylated Hp were optimized. We used as selection criteria the values of the S/B response ratios obtained in the presence of 1 ng mL^−1^ (total Hp immunoplatform) and 10 ng mL^−1^ (glycosylated Hp immunoplatform) of Hp standard (S) and in its absence (B). The amperometric transduction was performed under the previously optimized experimental conditions [29, 30]. It should be noted that although the Hp standard used is always the same, the different detector element involved in each bioplatform (Btn-Ab or SNA-FITC) is responsible for determining total or glycosylated Hp, respectively. The results of these studies are displayed in Figs. S1 and S2 of the Supplementary information and summarized in Table 1. Panels d and f of Figs. S1 and S2 compare the performance of different protocols used for the preparation of the immunoconjugates. All these protocols start from the immunoconjugates, CAb-MBs after ethanolamine blocking or Ab-Btn-Neu-MBs, for total or glycosylated Hp determination, respectively, and employ 30-min incubation steps in different solutions: 1 incubation step in a mixed solution containing Hp standard, detector bioreceptor (Btn-Ab or SNA-FITC for total or glycosylated Hp determination, respectively), and enzymatic tracer (Strep-HRP or Anti-FITC-HRP Fab fragments for total or glycosylated Hp determination, respectively) (1); 2 successive incubation steps: first in a mixed solution of Hp standard and detector bioreceptor and then in a solution of the enzymatic tracer (2i); 2 successive incubation steps: first in a solution of Hp standard and then in a mixed solution of detector bioreceptor and enzymatic tracer (2ii); and 3 successive incubation steps in solutions of Hp standard, detector bioreceptor, and enzymatic tracer, respectively (3).

**Table 1:** Tested and selected experimental variables for the preparation of immunoplatforms for the amperometric determination of total and glycosylated Hp

	Parameter	Tested interval	Selected value
Total Hp	MBs	Neutravidin or –COOH	–COOH
	[CAb], μg mL^−1^	0–50	25
	*t*_CAb_, min	0–60	15
	Buffer Hp	PBS, BB	BB
	Incubation steps	1–3	2
	[Btn-Ab], μg mL^−1^	0–2	0.05
	*t*_Hp + Btn-Ab_, min	0–60	30
	Strep-HRP dilution	1/50–1/5000	1/500
	*t*_Strep-HRP_, min	0–60	30
Glycosylated Hp	MBs	Neutravidin, –COOH or –COOH modified with APBA	Neutravidin
	Electrochemical measurement	Drop (TMB and HQ), solution (HQ)	Solution (HQ)
	[Btn-Ab], μg mL^−1^	0–1	0.1
	*t*_Btn-Ab_, min	0–60	30
	Buffer anti-FITC-HRP Fab fragments	PBS, BB or PBS/BB mixture	BB
	Incubation steps	1–3	3
	*t*_Hp_, min	0–60	30
	[SNA-FITC], μg mL^−1^	0–10	2.5
	*t*_SNA-FITC_, min	0–60	30
	[Anti-FITC-HRP Fab fragments], U mL^−1^	0–1	0.5
	*t*_Anti-FITC-HRP Fab fragments_, min	0–60	15

Among the results obtained in these optimization studies, a noticeable finding is the larger B-responses found for the determination of the glycosylated protein. This fact can be attributed to the nonspecific adsorption of SNA-FITC on the surface of the MBs, in agreement with that reported by other authors [10]. These nonspecific adsorptions are also evident in the determining role that the incubation medium plays in the S/B ratio (panel e, Fig. S2) and in the increase of B-responses with the increase in the concentration of the SNA-FITC solution or the corresponding incubation time (panels h and i, Fig. S2). In addition, they are probably also responsible for the fact that discrimination between the presence and absence of glycosylated Hp is only feasible when the working protocol involved independent incubation steps with the Hp, the detection bioreceptor, and the enzymatic tracer (panel f, Fig. S2).

**Fig. 2:**
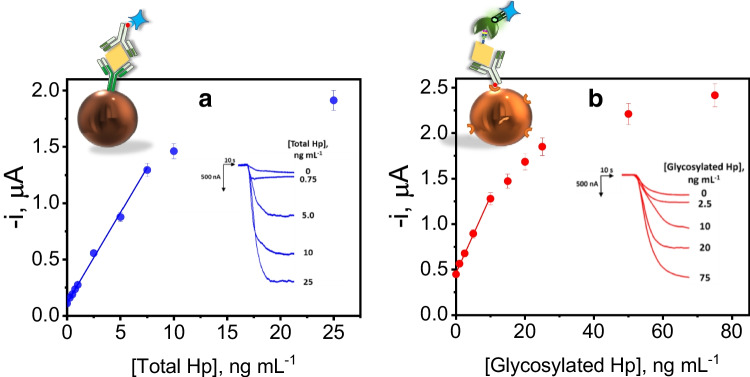
Calibration curves and amperometric traces recorded for the amperometric determination of Hp standards with the immunoplatforms developed for the determination of total Hp (**a**) and glycosylated Hp (**b**)

Once the experimental variables involved in the development of the bioplatforms were optimized, their analytical characteristics for the amperometric determination of Hp standards were evaluated. The resulting calibration curves are shown in Fig. 2, and the analytical characteristics summarized in Table 2.

**Table 2:** Analytical characteristics of the immunoplatforms developed for the single amperometric determination of total or glycosylated Hp

Parameter	Total Hp	Glycosylated Hp
Linear range, ng mL^−1^	0.25–7.50	1.5–10.0
Slope, nA mL ng^−1^	156 ± 7	82 ± 7
Intercept, nA	120 ± 20	470 ± 40
*R*^2^	0.9978	0.9977
LOD, ng mL^−1*^	0.07	0.46
LOQ, ng mL^−1**^	0.22	1.52
RSD, % (*n* = 10, 2.5 (total Hp) and 5.0 (glycosylated Hp) ng mL^−1^ Hp standard)	4.6	5.3
Analysis time, min^***^	60	75
Stability Ab-MBs (filtered PBS, 4 °C), days	8	0

^*^3×s_*b*_*/m*

^**^10×s_*b*_*/m*

^***^Starting from the blocked Ab-MBs or Ab-Btn-Neu-MBs

According to the data summarized in Table 2, the developed bioplatforms exhibited acceptable analytical characteristics, allowing the required determinations to be carried out in a maximum time of 75 min. The highest intercept value observed for the glycosylated Hp calibration plot must again be attributed to the nonspecific adsorption of SNA-FITC and thus of HRP molecules (anti-FITC-HRP Fab fragments) on the surface of MBs. The limits of quantification were between 10^5^ and 10^6^ times lower than the Hp levels found in the serum of healthy individuals (0.33 mg mL^−1^ [5]) or lung cancer patients (2 ± 1 mg mL^−1^ total Hp and 0.2 ± 0.1 mg mL^−1^ sialylated Hp [31]), which makes the developed immunoplatforms perfectly compatible with real clinical applications.

So far, the determination of total Hp has been carried out using different techniques involving colorimetric assays [32], surface plasmon resonance [33], chemiluminescence [34–36], surface-enhanced Raman scattering [37], or quartz crystal microbalance [38]. Additionally, some electrochemical biosensors for total Hp have been reported. Tan et al. developed an amperometric immunosensor for Hp determination in mastitic milk samples by immobilizing an anti-bovine Hp antibody on a gold nanostructured electrode. The immunosensor showed a linear relationship between 15 and 100 μg mL^−1^ and a LOD of 0.63 μg mL^−1^ [17]. A disposable electrochemical biosensor was built on an indium tin oxide substrate modified with aminopropyltriethoxysilane for the covalent immobilization of anti-Hp antibodies. Using electrochemical impedance spectroscopy, the determination of Hp was feasible over the 0.2 to 1.0 fg mL^−1^ concentration range, reaching a LOD of 0.001 fg mL^−1^ [18]. The determination of Hp in spiked human serum samples was carried out by square wave voltammetry within the 30 fg mL^−1^ to 1 ng mL^−1^ concentration range. This label-free immunosensor used the physisorption of a graphene nanoplatelets-chitosan nanocomposite on the surface of a glassy carbon electrode [20]. Tuteja and Neethirajan explored the use of phosphorene nanosheets for the electrochemical immunosensing of total Hp. This disposable phosphorene-based electrochemical immunosensor exhibited a dynamic linear concentration range from 0.01 to 10 mg mL^−1^ in standard and serum samples with a LOD of ∼ 0.011 mg mL^−1^ [19]. Another method used Hb-modified magnetic nanoparticles (Hb-MBs) for bovine Hp detection. Once Hp was complexed by the Hb-MB conjugates, it was captured with an anti-bovine Hp antibody further labeled with alkaline phosphatase. The method showed a range of detection from 25 to 800 μg mL^−1^ and a LOD of 43 ng mL^−1^ [21].

Importantly, the biosensor developed in our work shows better LOD (70 pg mL^−1^) than those claimed for most of the already reported methodologies for the determination of total Hp without requiring tedious protocols or complex and expensive instrumentation. Besides, most of the reported biosensors have only been applied to the determination of Hp in spiked samples, in contrast with our developed immunoplatform which has been employed for the determination of Hp in real samples at an affordable cost and with potential to be used in decentralized environments.

Regarding the determination of glycosylated Hp, Kazuno et al. developed a surface plasmon resonance (SPR) platform based on multi-sequential analysis using different lectins to estimate the glycosylation status of Hp in sera of patients with prostate cancer. The analysis involved the use of anti-Hp as ligand followed by the detection of Hp sugar chains by a lectin solution [11]. Nevertheless, to our knowledge, no electrochemical biosensing strategy has been reported for the determination of this glycosylated protein.

In addition, there are commercial ELISA kits available for total Hp determination. The ELISA tests are based on competitive or non-competitive immunoassays achieving worse (0.07 μg mL^−1^ [catalog number ab108856 from Abcam]) or similar sensitivities (31.2 pg mL^−1^ [catalog number: DY8465 from R&D Systems, Inc.], 86 pg mL^−1^ [catalog number ab219048 from Abcam]) than that of the developed immunoplatform. It is important to remark that these kits for Hp total determination mostly in human biofluids (serum, plasma, saliva, and urine) and some of them in cell culture supernatants (catalog number ab219048 from Abcam; Catalog Number: DY8465 from R&D Systems, Inc.) do not provide information on glycosylated Hp and require centralized instrumentation. In addition, the fastest analysis takes 90 min (catalog number ab219048 from Abcam).

The results summarized in Table 2 show a good reproducibility of the measurements performed with the single bioplatforms. The relative standard deviation (RSD) values calculated using 10 different immunoplatforms for the determination of total (RSD = 4.6%) and glycosylated (RSD = 5.3%) Hp demonstrate the robustness of the bioconjugate manufacturing processes and amperometric measurements.

Regarding the stability of the immunocaptors (stored, after their preparation, at 4 °C in filtered PBS), Fig. S3 in the Supplementary information shows as the immunocaptor used for the determination of total Hp (ethanolamine-blocked CAb-MBs) provided measurements within the control limits during 8 days (S/B for 2.5 ng mL^−1^ of Hp standard), those used for the determination of glycosylated Hp (Ab-Btn-Neu-MBs) show a progressive loss of sensitivity after their preparation (S/B for 5.0 ng mL^−1^ of Hp standard), which may be due to multiple factors (different types of MBs, antibodies, and immobilization strategy used for their fabrication).

The selectivity of the immunoplatforms was evaluated by comparing their responses for 2.5 (total Hp) and 5.0 (glycosylated Hp) ng mL^−1^ of Hp standards prepared in the absence and in the presence of potential interfering compounds, including other major serum proteins and cancer biomarkers. The obtained results (Fig. S4 in the Supplementary information) show that the presence of Hb and HSA (bars 2 and 3, respectively) affected the determination of both proteins, while the presence of IL-13Rα2 (bar 6) and IgG (bar 1) only affected the determination of total and glycosylated Hp, respectively. The interference of Hb can be attributed to both its endogenous peroxidase activity [39] and its affinity for Hp [8]. The nonspecific adsorption of IgG on MBs, and the possible coexistence of IgGs when working with a not highly purified protein standard, in the case of HSA [40], may explain the observed interference. However, it should be noted that the presence of these proteins affects mainly in the analysis of serum. Furthermore, as it is shown in Fig. S4 (bars 1B, 2B, 3B, and 6B), a large dilution of the samples avoids such interferences. Such dilution is feasible considering the high sensitivity achieved with the developed immunoplatforms.

### Simultaneous determination of total and glycosylated Hp

After the bioplatforms were prepared and characterized for the single determination of total and glycosylated Hp, disposable platforms for dual and quadruple detection were tested for multiplexing purposes. According to the results shown in Fig. S5 in the Supplementary information, the SP_4_CE platform provided similar *S*/*B* ratios, for intermediate concentrations of the Hp standard, which were obtained with the single detection platforms. Since the employed magnetic bioconjugates are the same and the amount of them captured is also the same, this behavior should be attributed to the slightly larger area of the working electrodes in the quadruple platforms compared to the dual ones (6.8 vs. 6.3 mm^2^) and especially to their shape (circular vs. ellipsoidal) and electrode distribution, which is more like that of the single detection platforms (12.6 mm^2^). According to these results, quadruple detection platforms were selected for multiplexed analyses.

Table S1 in the Supplementary information compares the analytical characteristics of the calibration plots constructed with the single and quadruple detection platforms for the amperometric determination of total and glycosylated Hp. As it can be seen, no large differences in the key analytical parameters were found, thus supporting the choice of the multiplexed platform to evaluate the applicability of the developed methodologies.

### Application to the simultaneous determination of total and glycosylated Hp in secretomes from cell culture conditioned media

**Fig. 3:**
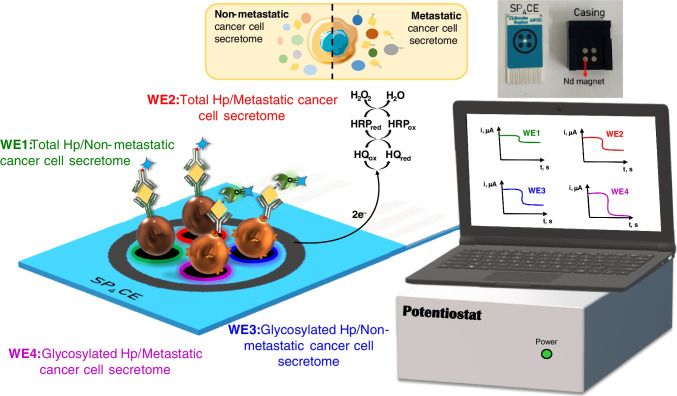
Schematic display of the quadruple immunoplatform developed for the simultaneous determination of total and glycosylated Hp in the secretome of metastatic and non-metastatic CRC cells

The applicability of the quadruple detection bioplatforms for the analysis of the secretomes of in vitro–cultured CRC cells with different metastatic potential (Fig. 3) was evaluated.

The results obtained in the evaluation of the possible existence of matrix effect (Table S2 in the Supplementary information), and considering the endogenous content of total and glycosylated Hp in the secretomes, led us to use the standard addition method with undiluted secretomes and additions between 0 and 5 ng mL^−1^ of the Hp standard, for the determination of total Hp. The determination of glycosylated Hp was accomplished by analyzing fivefold-diluted secretomes and using the external standard calibration in the case of KM12 cells secretomes, and the standard addition method for SW cell secretomes. In all cases, the calibrations included a range of Hp standard concentrations between 0 and 5 ng mL^−1^.

The concentrations obtained using the developed quadruple immunoplatform for total and glycosylated Hp in the different secretomes are summarized in Table 3. It is important to note that the reported concentrations were corrected by subtracting the small non-specific signal that comes from the medium in which the cells are grown (DMEM).

**Table 3:** Endogenous total and glycosylated Hp contents and recovery percentages (mean value ± ts/√*n*; *n* = 3; *α* = 0.05) obtained in the secretome samples of CRC cells with the developed quadruple immunoplatform

Cells	Total Hp			Glycosylated Hp		
	[Total Hp], ng mL^−1*^	RSD, %	Recovery, %^**^	[Glycosylated Hp], ng mL^−1*^	RSD, %	Recovery, %^**^
KM12C	0.31 ± 0.03	3.8	99 ± 7	10.1 ± 0.3	1.1	102 ± 2
KM12SM	0.37 ± 0.06	6.4	100 ± 20	13 ± 3	9.2	103 ± 3
KM12L4a	0.6 ± 0.1	8.4	100 ± 20	14 ± 2	4.3	102 ± 3
SW480	0.28 ± 0.02	3.4	110 ± 20	12 ± 2	5.6	95 ± 5
SW620	0.50 ± 0.05	4.3	100 ± 20	16 ± 4	9.0	102 ± 4

^*^Endogenous concentrations found

^**^% recovery values obtained once the secretomes were enriched with 1 ng mL^*–1*^ *of total Hp standard and the previously determined endogenous contents subtracted from the total concentration*

The results summarized in Table 3 confirm the upregulation of total and glycosylated Hp in the secretome of cells with higher metastatic potential (KM12SM, KM12L4a, and SW620). The fact that the concentration of glycosylated Hp was higher than that of total Hp may be attributed to the hyperglycosylation of Hp in tumor cells, which prevented the recognition of total haptoglobin by epitope masking because the antibodies recognize primarily the peptide region of the glycoprotein and not the glycans. These results can also be justified by the fact that the use of a lectin as a detection element and a highly glycosylated target protein, “global” information on the level of Hp glycosylation was obtained.

**Fig. 4:**
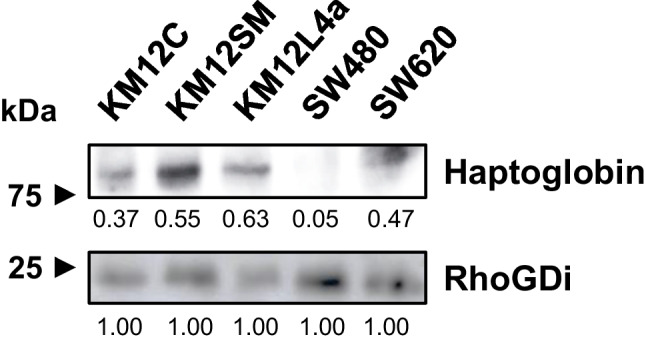
Western blot analysis of total Hp in CRC cell protein extracts (10 μg) using RhoGDi as loading control revealed. ImageJ was used for semi-quantification of the protein bands of Hp and RhoGDi. Protein bands were normalized using RhoGDi

The quantification of total and glycosylated Hp in the secretomes was not possible using the commercial ELISA kit (Ref DY8465 – R&D Systems and developed for the determination of total Hp in most cell culture supernatants, urine, saliva, plasma, and serum) or the WB methodology (due to lack of sensitivity). Nevertheless, the results provided by the bioplatforms for total Hp in the secretomes agree with the semi-quantitative results obtained by WB in cell protein extracts and normalized using RhoGDi (Fig. 4). As can be seen, higher levels of total Hp were obtained in cell protein extracts of metastatic CRC cells (KM12SM, KM12L4a, and SW620) in comparison to their isogenic but non-metastatic CRC cell counterparts (KM12C and SW480), respectively.

Recovery studies were carried out to evaluate the accuracy of the results obtained in the analysis of secretomes from CRC cells. The studies were performed by analyzing the different secretomes supplemented with 1 ng mL^−1^ of the Hp standard using the same protocols employed for the determination of the endogenous contents. The recovery values summarized in Table 3 proved the reliability of the developed quadruple immunoplatforms for the analysis of total and glycosylated Hp in these particularly challenging samples, due to the low concentrations of the target proteins. Moreover, the obtained results allow the possibility of discriminating the expressed secretome of homologous CRC cells with different invasive/metastatic potential, which is highly important to understand their different molecular mechanism of invasion and metastasis [41], through the simple determination of the secreted content of both target proteins in the media in which these cells have been grown.

It should be emphasized that the good selectivity and sensitivity exhibited by the developed bioelectronic immunoplatforms make them potentially useful for quantitative secretome analysis of cancer cells either in serum-free (such as those analyzed in this study) or in serum-containing conditioned medium, ensuring in both cases that the state of the cells was not altered in the presence or absence of serum [42].

## Conclusions

In this work, we propose the first electrochemical immunoplatforms for the single or simultaneous determination of total and/or glycosylated Hp. The immunoplatforms use magnetic immunocaptors and another antibody or a lectin, as detector elements, respectively. The resulting bioconjugates are enzymatically labeled with the HRP enzyme and captured for amperometric transduction on disposable electrochemical substrates for single or multiplexed detection. Under the optimized experimental conditions, the immunoplatforms exhibit analytical and operational characteristics compatible with their practical applicability. Moreover, the potential of the immunoplatforms for multiplexing has been demonstrated for the determination of endogenous total and glycosylated Hp content in the secretomes from CRC cells with different metastatic potentials in approx. 75 min. It is worth noting the absence of commercially available ELISA kits for the determination of glycosylated Hp and the lack of sensitivity of WB methodologies for the determination of total Hp in the prepared secretomes. The reliability and accuracy of the results provided by the developed immunoplatforms have been verified by performing recovery studies in the same secretomes and by the similar behavior obtained by WB in the cell protein extracts of the five types of CRC cells. It is important to highlight the versatility of the developed bioplatforms that can be easily adapted to the determination of other glycoproteins just by preparing immunocaptors with the corresponding selective antibodies because the used lectin should serve to detect glycosylation in any altered glycoprotein in cancer. The developed immunoplatform can be employed to provide a more reliable diagnosis of the disease and its stage through the simultaneous determination of the glycosylated and non-glycosylated target protein.

## Supplementary Information

ESM 1 (DOCX 443 kb)

## Data Availability

The data that support the findings of this study are available from the corresponding authors upon reasonable request.

## Declarations

### Disclaimer

The funders had no role in the design of the study; in the collection, analyses, or interpretation of the data; in the writing of the manuscript; or in the decision to publish the results.
