# Supplementary material for: First bioelectronic immunoplatform for quantitative secretomic analysis of total and metastasis-driven glycosylated haptoglobin
Source: Anal Bioanal Chem. 2022 Nov 8;415(11):2045–57. doi: 10.1007/s00216-022-04397-6 (PMC10079713; doi:10.1007/s00216-022-04397-6)
Supplement: Supplementary file 1 — (DOCX 443 kb) [file 216_2022_4397_MOESM1_ESM.docx]

**SUPPLEMENTARY INFORMATION**

**First bioelectronic immunoplatform for quantitative secretomic analysis of total and metastasis-driven glycosylated haptoglobin**

Cristina Muñoz-San Martín,^1^ Ana Montero-Calle,^2^ María Garranzo-Asensio,^2^ Maria Gamella,^1^ Víctor Pérez-Ginés,^1^ María Pedrero,^1^ José M. Pingarrón,^1^ Rodrigo Barderas,^2,*^ Noemí de-los-Santos-Álvarez,^3^ María Jesús Lobo-Castañón,^3,*^ Susana Campuzano^1,*^

^1^Departamento de Química Analítica, Facultad de CC. Químicas, Universidad Complutense de Madrid, 28040 Madrid, Spain

^2^UFIEC, Instituto de Salud Carlos III, 28220 Majadahonda, Madrid, Spain.

^3^Departamento de Química Física y Analítica. Universidad de Oviedo, 33006 Oviedo; Instituto de Investigación Sanitaria del Principado de Asturias, 33011 Oviedo, Spain

** to whom correspondence should be addressed (r.barderasm@isciii.es,* [*mjlc@uniovi.es*](mailto:mjlc@uniovi.es)*,* [*susanacr@quim.ucm.es*](mailto:susanacr@quim.ucm.es)*)*

| **CONTENTS** | **PAG.** |
| --- | --- |
| **Fig. S1** | S2 |
| **Fig. S2** | S3 |
| **Fig. S3** | S4 |
| **Fig. S4** | S4 |
| **Fig. S5** | S5 |
| **Table S1** | S5 |
| **Table S2** | S5 |

**Fig. S1** Optimization of experimental variables for the determination of total Hp. Influence of the indicated variable on the amperometric responses provided by the resulting immunoplatform for the single determination of total Hp in the presence of 1 ng mL^–1^ of Hp standard (S, grey bars) and in its absence (B, white bars). The values of the corresponding S/B ratios are shown in blue color

**Fig. S2** Optimization of experimental variables for the determination of glycosylated Hp. Influence of the indicated variable on the amperometric responses provided by the resulting immunoplatform for the single determination of glycosylated Hp in the presence of 10 ng mL^–1^ of Hp standard (S, grey bars) and in its absence (B, white bars). The values of the corresponding S/B ratios are displayed in red color

**Fig. S3** Storage stability of the immunocaptors, ethanolamine-blocked CAb-MBs or Ab-Btn-Neu-MBs, involved in the amperometric determination of total a) and glycosylated b) Hp, respectively. Control limits (dashed lines) were set as ± 3 s of the S/B average value obtained with the immunoconjugates prepared in the day zero (n = 3)

**Fig. S4** Effect of the presence of potential interferents on the amperometric responses provided by the developed immunoplatforms for the determination of total a) and glycosylated b) Hp in the presence of 0.0 (white bars) and 2.5 (total Hp) and 5.0 (glycosylated Hp) ng mL^−1^ (grey bars) of Hp standards prepared in the absence (0) and in the presence of: 1 mg mL^–1^ IgG (1/1A); 0.01 mg mL^−1^ IgG (1B); 5 mg mL^−1^ Hb (2A); 0.05 mg mL^−1^ Hb (2B); 50 mg mL^−1^ HSA (3A); 5×10^−5^ mg mL^–1^ HSA (3B); 10 ng mL^−1^ TNF-α (4); 500 ng mL^−1^ CDH-17 (5); 50 ng mL^−1^ IL-13Rα2 (6/6A); 5 ng mL^−1^ IL-13Rα2 (6B)


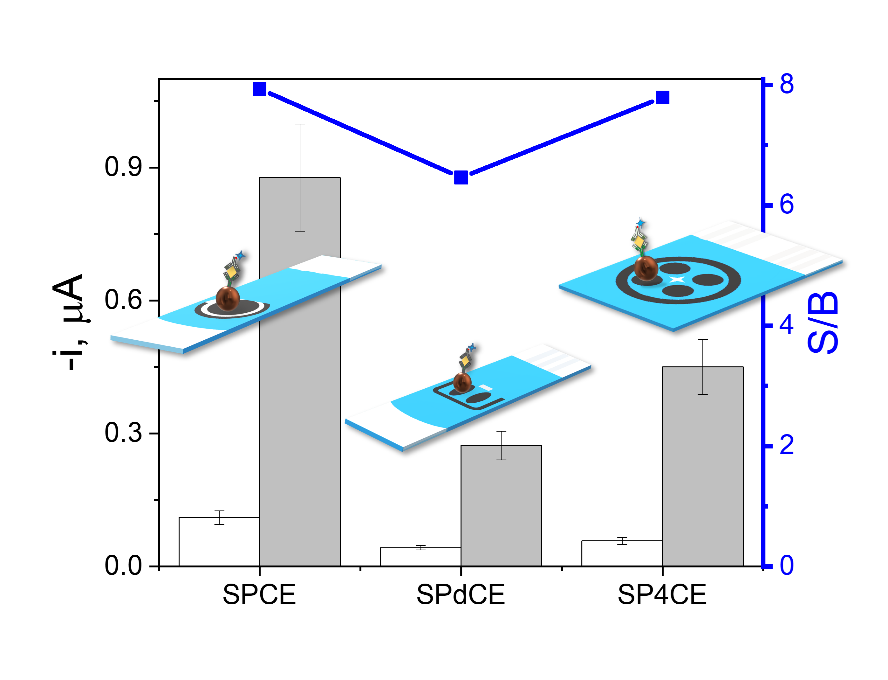


**Fig. S5** Comparison of the amperometric responses obtained in the absence and in the presence of 5.0 ng mL^−1^ Hp with the bioplatforms constructed using SPCEs, SPdCEs and SP_4_CEs

**Table S1** Comparison of the analytical characteristics obtained for the amperometric determination of Hp standards with bioplatforms constructed using SPCEs or SP_4_CEs

|  | **Total Hp** | | **Glycosylated Hp** | |
| --- | --- | --- | --- | --- |
| **Parameter** | **SPCEs** | **SP_4_CEs** | **SPCEs** | **SP_4_CEs** |
| Linear range, ng mL^–1^ | 0.25 – 7.50 | 0.25 – 7.50 | 1.5 – 10.0 | 1.0 – 10.0 |
| Slope, nA mL ng^–1^ | 156 ± 7 | 79 ± 4 | 82 ± 7 | 120 ± 10 |
| Intercept, nA | 120 ± 20 | 60 ± 20 | 470 ± 40 | 700 ± 60 |
| R^2^ | 0.9978 | 0.9987 | 0.9977 | 0.9970 |

**Table S2** Comparison of the slope’s values obtained (in nA mL ng^−1^) with the immunoplatforms developed for the determination of total and glycosylated Hp in the different media tested

| **Media** | **Total Hp** | **Glycosylated Hp** |
| --- | --- | --- |
| Buffered solutions | 79 ± 4 | 120 ± 10 |
| KM12 cells secretomes | 82 ± 4 | 120 ± 10 |
| SW cells secretomes | 37 ± 2 | 50 ± 4 |
